# Qualitative Inquiry into Challenges Experienced by Registered General Nurses in the Emergency Department: A Study of Selected Hospitals in the Volta Region of Ghana

**DOI:** 10.1155/2016/6082105

**Published:** 2016-11-03

**Authors:** Confidence Alorse Atakro, Jerry Paul Ninnoni, Peter Adatara, Janet Gross, Michael Agbavor

**Affiliations:** ^1^University of Cape Coast, Cape Coast, Ghana; ^2^Faculty of Nursing and Midwifery School, University of Cape Coast, Cape Coast, Ghana; ^3^The School of Nursing and Midwifery, University of Health and Allied Sciences, Ho, Ghana; ^4^Morehead State University, Morehead, KY, USA; ^5^Global Health Services Partnership, US Peace Corps, Washington, DC, USA; ^6^Volta Regional Hospital, Ho, Ghana

## Abstract

Registered General Nurses (RGNs) play crucial roles in emergency departments (EDs). EDs in Ghana are primarily staffed by RGNs who have had no additional formal education in emergency care. Additionally, basic, master's, or doctoral level nursing education programs provide limited content on the complexities of emergency nursing. Nurses in EDs are affected by many challenges such as growing patient population, financial pressures, physical violence, verbal abuse, operational inefficiencies, overcrowding, and work overload. There is a paucity of research on challenges experienced by RGNs in EDs in the Volta Region of Ghana. In this qualitative study, twenty RGNs in EDs from three selected hospitals in the Volta Region of Ghana were interviewed. All recorded interviews were transcribed, reviewed several times by researchers and supervisors, and analyzed using content analysis. Five thematic categories were identified. These thematic categories of challenges were lack of preparation for ED role, verbal abuse from patients relatives, lack of resources in ED, stressful and time consuming nature of ED, and overcrowding in ED. Formal education of RGNs in the advanced role of emergency care, adequate supply of resources, increased hospital management support, and motivations for RGNs working in ED are necessary to improve the practice of emergency care.

## 1. Introduction

Emergency care is care that must be rendered without delay [1]. The task of emergency departments (EDs) is to provide safe emergency health care to all who need it while adopting a caring, cost-effective approach [2, 3]. Nurses are frontline workers in EDs [4] and experience challenges of overcrowding, growing admission volumes, inadequate resources, and operational inefficiencies in ED [5, 6]. The actual situation in an ED is that when the number of ED patients increases or patients with more severe conditions are admitted to an ED, individual demands for direct nursing care increases; therefore, patients' needs often cannot be met by the same number of nursing staff members [7]. Nursing staff are forced to reduce contact time with patients [7]. Reducing contact time with patients poses threats to the provision of quality nursing care to clients in EDs [7]. Challenges experienced by RGNs working in ED are aggravated by leadership issues and inadequate preparations for ED roles [8]. The scope of practice for emergency nurses in Africa is limited with no clearly defined advanced nurse practitioner roles [9]. Despite the fact that care provided by nurse practitioners practicing in ED requires a body of knowledge relating to acute illness and injury [9], there are few institutions that offer emergency nurse training in Africa and, therefore, few emergency nurses are available to provide care in EDs [10]. While emergency medicine training programs for physicians are on the rise, there are few established training programs for emergency nurses [11]. Caring for emergency patients in Africa is particularly challenging because nurses must often treat severely injured patients who have coexisting conditions, such as Human Immunodeficiency Virus (HIV), Acquired Immunodeficiency Syndrome (AIDS), or Tuberculosis (TB) [10]. The presence of HIV, AIDS, and TB complicates client care and has health implications for staff themselves [10]. Nurses working in EDs in Africa also work with limited resources [10]. Most emergency departments in Africa are staffed by RGNs with no additional formal education in emergency nursing [10]. In Ghana, as in other developing and middle-income countries, little consideration has traditionally been given to optimising the training of nursing staff for the care of acutely ill or injured patients [12]. Additionally, existing emergency care systems in Ghana are rudimentary in comparison to those in developed countries [12] aggravating an already bad situation of overcrowding and inadequate preparations of nurses in emergency care [13]. Though RGNs in EDs are challenged in the provision of safe, quality care to clients, ED nurses' experiences of challenges within accident and emergency units are seldom investigated [14]. Knowledge of challenges experienced by RGNs in ED is necessary to address challenges in EDs. The purpose of this study was to explore challenges experienced by RGNs working in EDs in selected hospitals of the Volta Region of Ghana.

## 2. Materials and Methods

A qualitative phenomenology study design was used in carrying out this study. Phenomenology, rooted in a philosophical tradition, is an approach to explore people's everyday life experiences [15]. The qualitative study design was chosen to explore challenges experienced by RGNs in emergency departments in selected hospitals of the Volta Region of Ghana.

### 2.1. Setting

The settings for data collection were three public hospitals in the Volta Region of Ghana that had emergency departments. These hospitals were Volta Regional Hospital, Keta Municipal Hospital, and Ho Municipal Hospital.

### 2.2. Study Population

The target population for this study was RGNs working at the ED in the Volta Regional Hospital (VRH), Keta Municipal Hospital (KMH), and Ho Municipal Hospital (HMH) in the Volta Region of Ghana.

### 2.3. Data Collection and Analysis

Participants for data collection were selected through purposive sampling technique. Data were collected in January 2015, within a three-week period. A list of all RGNs in EDs was requested from Nurse Managers of EDs. Inclusion criteria were RGNs with professional identification numbers (PINs) who had worked for one year or more and were willing to give written consent. The eligible sample size was estimated to be 20 participants, large enough for data saturation. Saturation was reached after interviewing 20 participants, when new data confirmed previous data without adding new insights [16]. The mobile phone numbers of selected participants were requested. Participants were called on phone to arrange a convenient time and place for interviews. As no validated interview guide relevant to the topic of interest was available, the themes of the interview and the questions were developed by the research group, based on previous scientific literature. EDs were visited prior to data collection to present the study and inform the staff about the study. Staff had opportunity to ask questions and receive answers concerning the study. One semistructured interview was conducted with each participant. Interviews took place at places convenient for participants. Interviews were audio-recorded and recordings were transcribed verbatim. Participants told their stories with minimal interruption. The interviews ranged from 30 to 60 minutes. Transcribed interviews were stored in electronic folders that were created and labeled appropriately for easy identification. These folders were kept on a pen drive solely meant for the purpose of the study and kept under lock and key. Transcribed data were analyzed using conventional qualitative content analysis. Each transcribed interview was read several times and the primary codes were extracted. Then, the related codes were put in groups. Categories were developed based on similarity and content of codes. To ensure trustworthiness of data, continuous investigation of the data (transcription of the data and investigating until the main themes were obtained), peer check and member check were performed. Moreover, the objectivity of the data was determined through continuous, accurate, and proper treatment of all stages of the research study and clarity of the research method. Also, using a team approach in data analysis (i.e., reviewing the data analysis by the research team), the reliability of the results was confirmed. Themes developed were (a) lack of preparation for ED role, (b) verbal abuse from patients' relatives, (c) lack of resources in ED, (d) stressful and time consuming nature of ED, and (e) overcrowding in ED.

### 2.4. Ethical Considerations

The research proposal was submitted to and approved by the University of Cape Coast Institutional Review Board (UCCIRB). Additionally, study received approval from each hospital where research was conducted. Adherence to all principles of research ethics was strictly observed. Participants were briefed about the study aim and procedures before written informed consent was obtained from them. Participants were informed about their rights to refuse to participate in the study or to leave at any time without giving any reason. Also, participants were informed that their refusal to participate in the study would not be used against them in any form. Confidentiality of participants was enforced, and they were assured that the data would be used only for research purposes. The study process did not entail any harmful effects on participants.

## 3. Results and Discussions

### 3.1. Results

In this study, 75% of respondents were between 25 to 29 years of age. A majority of RGNs working at the emergency department (90%) studied general nursing at the Nurses' Training College (NTC). Only 5% of RGNs had additional knowledge of critical care nursing (an advanced diploma course in critical care nursing) to practice emergency care in ED. None of the respondents studied emergency nursing as a degree program. Seventy percent (70%) of participants had worked for two years or less in ED. As many as 75% of participants indicated that there was only one professional nurse in a shift in ED. Five thematic categories were extracted after data analysis. The categories of challenges experienced by RGNs working in ED in selected hospitals in the Volta Region of Ghana were lack of preparation for ED role, verbal abuse from patient relatives, lack of resources in ED, stressful and time consuming nature of ED, and overcrowding in ED.

#### 3.1.1. Lack of Preparation for ED Role

Almost all RGNs interviewed considered lack of preparation for ED role as a challenge in ED. One participant likened ED to a battle ground.

*Nurses are just brought into the emergency unit to battle like soldiers who don't even know how to shoot guns. You have to push yourself to learn because there is no emergency nurse role models to learn from. [participant 7]*

The majority of participants described how the unavailability of emergency nurses and physicians in ED affected their learning experiences. A participant described this in the following statement:

*ED exposes you to so many conditions but there are no emergency nurses or emergency physicians in ED. I have to force myself to learn. Sometimes I try something to see whether it will work. It's like lotto which is not good enough. [Participant 2]*

Many participants reported that there were many deaths in ED which could have been prevented by the presence of well-educated emergency nurses and physicians. This was evident in the words of a participant.

*Working in ED is emotional because patients die easily. I think if there were trained emergency nurses and physicians, we could prevent some of the deaths. I feel sad when people die like that.*

Many participants wished that, in the absence of formal education in ED practice, they could be taken through some emergency care workshops before placement in the emergency care unit. A participant explains this in the following statement:

*We are all RGNs and have no specialist education in emergency care. At least before one is placed in ED, He or she should be taken through some training in the form of a workshop so that he or she will be ready for ED nursing. [participant 14]*

#### 3.1.2. Verbal Abuse from Relatives toward ED Nurses

Most of the participants in this study considered verbal abuse from relatives toward ED nurses as another challenge in ED. In this regard a participant stated the following:

*Relatives here sometimes are ungrateful. Some relatives insult and talk to you very rudely. [participant 10]*

Another participant also said the following:

*There is stress in ED and patient relatives don't appreciate. Sometimes you are exhausted and the patient and relative coming in don't know and always want immediate attention. If they don't get it, they insult you. [participant 4]*

Another participant explained his/her frustration with patient relatives:

*Here, relatives want to tell us what emergency care should be. They always put pressure on you to take care of their relative even if it's not an emergency. They verbally abuse you when they feel you are not taking care of them the way they want. [participant 6]*

#### 3.1.3. Lack of Resources in ED

Another category of challenges extracted from interviews was lack of resources in ED. In this regard a participant stated:

*“The supply of essential materials is not the best. Sometimes supply of materials delay and you are embarrassed when clients come and you cannot care for them as a result of lack of materials. We also need more doctors and nurses in ED [participant 19]”.*

Moreover, one other participant stated the following:

*The problem is about staffing. At least staff strength should be increased. Nurses are really sacrificing in ED. We do not have items too and patients come and it looks like we are not prepared for them. [participant 7]*

#### 3.1.4. Stress and Time Consumption Nature of ED

Stress and time consuming nature of ED was the fourth challenge in ED that participants described. The majority of respondents indicated that ED was stressful and time consuming. A participant described this in the following statements:

*I do extra hours on duty but am not motivated in any way. I do not partake in church activities as I used to do. Sometimes friends call me but I am not able to call back because of tiredness. [participant 7]*

Yet one other participant stated the following:

*Sometimes you don't have time for yourself or even your family, because of the staff situation. Social life is affected. You can't go for occasions. [participant 1]*

A participant also described the stress in ED in the following statement:

*It's like going to work and never sitting your ass down, working, going up and down. Sometimes you leave work late because you have to assist colleagues who have come to work. [participant 2]*

#### 3.1.5. Overcrowding in ED

Participants said that lack of space was a major challenge experienced in ED. In this regard one of the study participants said the following:

*Accident and Emergency unit is always hectic. We always add extra stretchers. The place is always messy. [participant 1]*

One other participant believed that there was always pressure on nurses in ED as a result of ED overcrowding. This is described in the following statement:

*There is so much pressure over here and we are not organised. And if you are not careful, you may give the wrong medication to a client. Sometimes we misplace clients medications as well. The lack of space and disorganisation makes the pressure worse. [participant 12]*

One participant explains reasons for overcrowding in the ED in the following statement:

*This place is choked with clients because the place is too small and also other wards do not want to accept stabilised cases. They will always tell you they don't have beds meanwhile when you go there you will see so many empty beds. [participant 19]*

## 4. Discussion

The findings of this study showed a clear picture of challenges experienced by RGNs in ED. The study participants stated their experiences in ED through five thematic categories.

Almost all nurses taking part in this study mentioned lack of formal education in emergency care as a challenge in emergency care and believed formal education in emergency care will help prevent unnecessary deaths in ED. Additionally, sociodemographic results show that only 5% of participants had specialist education to practice emergency nursing [17] Graneheim and Lundman showed that while emergency medicine training programs for physicians are on the rise, there are few established training programs for emergency nurses [16]. Little time is spent in teaching or learning the content of emergency nursing during the basic, master's, or doctoral level nursing education programs [8]. Caring for the acutely ill or injured patient requires multiple disciplines and specialists working seamlessly together; this care is a team-based continuum and does not end with the Emergency Physician [12]. In spite of the importance of formal education in emergency care, little consideration has been given to optimising the education of nursing staff for the care of acutely ill or injured patients in Ghana [12]. It has been estimated that persons with life-threatening but salvageable injuries were six times more likely to die in Ghana than in the United States of America [12]. Mishandling of severely injured patients by untrained persons, inadequately trained staff, and inadequate equipment were reported as contributory factors to the high mortality rates of ED clients in Ghana [12]. Emergency nursing as a profession is very new to Africa and therefore the availability of guidelines and standards is also limited [17]. Improvements in training such as developing an education curriculum for staff in all areas of the emergency care, holding in-service training on protocols for triage, and emergency care could improve nursing care in ED [18]. There should be enough protocols to help nurses deal with emergency cases in EDs. Management of health facilities within the Volta Region should also have plans of sponsoring nurses to pursue emergency nursing as a specialist course so that these emergency nursing specialists can return to provide leadership and mentorship in the EDs. Unit heads of EDs should organise frequent internal workshops on emergency nursing for ED nurses to upgrade their knowledge in emergency care. Resource person such as emergency physicians, critical care nurses, nurse anesthetists, and emergency care nurses should be invited from teaching hospitals to educate nurses at the EDs in a workshop. Orientations in ED in a form of workshop will improve the readiness of nurses to work in EDs. An Emergency Nursing Association could be formed by the few emergency and critical care nurses in Ghana to push the agenda of the education of more emergency care nurses in Ghana.

Another important dimension of challenges experienced by nurses in the emergency department was verbal abuse from relatives. Participants mentioned that patient relatives were sometimes aggressive toward them and verbally abused them. Participants indicated that, most of the time, relatives of clients want to tell nurses what constituted emergencies. These situations make ED a vulnerable setting for workplace violence [19]. Violence against ED nurses is prevalent [19]. A study conducted in the United States of America (USA) to investigate emergency nurses' experiences and perceptions of violence from patients and visitors in EDs found that approximately 25% of respondents experienced physical violence at least 20 times in 3 years [19]. Similarly almost 20% reported experiencing verbal abuse more than 200 times during the same period [19]. The main perceived reasons for violence were overcrowded emergency rooms, long waiting times, and inadequate systems of security [20]. Respondents who experienced frequent physical violence and/or frequent verbal abuse indicated lack of support from hospital administration and ED management in dealing with abuses [19]. A similar study conducted in South Africa by Kennedy and Julie concluded that the absence of policies to deal with ED violence contributed to underreporting [21]. There is need for hospital management support and policies that deal with emergency care abuses by patient relatives. A preventive, risk-management approach that addresses policies, restraint, security arrangements, and legal precedents is particularly important in managing ED violence [22]. Identification of trends and patterns of violence in ED is necessary so that better health care planning and service provision as well as effective preventive and safe strategies for nurses in the ED workplace can be implemented [23]. Management in hospitals in the Volta Region could organise educative programs on local radio stations and in churches or mosques in order to make the public know about challenges and processes in emergency care. This may reduce the verbal attacks on nurses from patient relatives.

Lack of resources in ED was a challenge participants thought must be addressed for the provision of quality emergency nursing care. RGNs working in ED said there were inadequate material and human resources to provide timely care to clients in ED. Nurses usually felt embarrassed when clients reported at the EDs without the necessary available resources to care for them. This is in agreement with Hines et al. [24] who indicated that nurses working in ED face a number of challenges which include decreased reimbursement by insurers and fewer health care resources including medical staff, facilities, adequate financing, and modern technologies [24]. In most emergency units in Ghana, patients presenting are not triaged and most emergency centres are poorly equipped and overcrowded [12]. Existing emergency care systems in Ghana are rudimentary in comparison to those in developed countries [12]. An assessment of an ED at Police Hospital, a second-level hospital in Accra, Ghana, revealed marked deficiencies in many essential items and services [18]. Nurses in Ghana are currently an underdeveloped resource for the provision of high quality emergency care [13]. However, the future of emergency care can be bright with support from hospital management, government, and international partners [12]. EDs should be allocated more material and human resources by hospital managements to be able to deal with the high patient acuity of EDs.

Another important category of challenges in emergency care was stress and time consuming nature of ED. Nurses on emergency wards indicated that they worked overtime without any motivations. Stress and time consuming nature of ED prevented some nurses from participating in social activities such as weddings and funerals. Emergency work is filled with unanticipated situations and is complicated by disturbed rest periods, long working hours, and limitations in staffing levels [25]. These factors tend to affect RGNs in EDs negatively [25]. RGNs in EDs work in a charged atmosphere that is overloaded with sensory stimuli (ringing phones, rushing people, and beeping monitors), all in a framework of urgency [26]. The quietest day in ED may suddenly become extremely hectic [26]. Rapid disposition of patients to other wards may be necessary to make space for patients in more critical condition [26]. Recent studies have shown that occupational stress is much greater for nurses who work within EDs [27]. A study carried out by Adeb-Saeedi, to identify sources of stress for nurses working within the ED of teaching hospitals, identified dealing with patients' pain and suffering, heavy workload, and the presence of the patients' family in the ED as sources of stress reported by RGNs [27]. Other factors identified as stressors were physical working environment and lack of staff [27]. A study was conducted by Ross-Adjie et al. to determine which stress-evoking incidents in Western Australian emergency department nurses perceive as most significant [28]. Violence against staff was the top ranked stressor, with workload as second [28]. Dealing with a mass casualty incident, the death/sexual abuse of a child, and dealing with high acuity patients were all closely ranked as third, fourth, and fifth, respectively [28]. Nurses working in EDs stated that debriefing after stress-evoking incidents in the workplace should be mandatory and should be conducted by professionals with specific debriefing and counseling skills [28]. Nurses also suggested other ways in which occupational stress could be reduced, including employing more staff and providing a “time-out” room [29]. It is suggested that while it may not be possible to decrease the demands of the ED nursing care, improving work conditions and providing greater support by hospital management to nurses may assist in decreasing the stressors associated with working in the EDs [27]. Currently no motivations exist for nurses working in EDs in VRH, KMH, and HMH. Nurses in EDs should be motivated by managements of EDs for overtime work. Motivations could be in the form of extra duty allowances or food for lunch.

Findings from this study also confirmed overcrowding as a challenge RGNs working in EDs experienced in the Volta Region. All participants confirmed the necessity to create enough space at the ED to make it easier to care for clients. Many of the participants see ED as a messy or disorganised environment. In most emergency units in Ghana, patients presenting are not triaged and most emergency centres are poorly equipped and overcrowded [12]. This is consistent with Coughlan and Corry who conducted a study in which participants described the emergency departments as resembling a disaster zone or a hospital scene from a third-world country [30]. Causes of ED overcrowding included hospital bed shortages, high medical acuity of patients, increasing patient volume, limited examination spaces, and shortage of staff nurses [31]. Overcrowding results in death, permanent disability, additional procedures, and increased length of hospitalization in EDs [31]. A study of California EDs found that 90% had overcrowded conditions and that overcrowding was present in both urban and rural areas [31]. The inability to provide timely service results from overcrowded conditions and has been implicated in poor outcomes for patients [31]. Although overcrowding has been the topic of discussion among many emergency health personnel, few scientific studies actually document and analyze the problems [31]. Despite dramatic headlines and photos of congested EDs, overcrowding has been largely ignored by governments and health care policy makers [31]. A survey by Richardson et al. showed that overcrowding is perceived to be a serious problem by emergency department directors [32]. Most of the factors that contribute to overcrowding are beyond the control of emergency departments [32]. ED overcrowding has multiple effects, including prolonged pain and suffering, long patient waits, patient dissatisfaction, decreased nurse and physician productivity, increased frustration in medical staff, and violence [31]. Solving the problem of overcrowding will not only require a major financial commitment from government and local hospitals, but will also require cooperation from management of hospitals [31]. Nurses in other wards should be educated on the challenges of overcrowding in ED so that they will be willing to accept cases transferred to them from EDs in order to create space for more acute cases in EDs.

Unless the problems in EDs are solved in the near future, the general public may no longer be able to rely on EDs for quality and timely emergency care, placing the people of this country at risk [31].

## 5. Conclusion

Though RGNs play crucial roles in EDs, they experience many challenges that are seldom investigated. It is important for stakeholders of EDs such as government, nongovernmental organisations (NGO), hospital managements, corporate organisations, and local authorities to invest attention and resources into EDs to reduce challenges in EDs. Steps that could improve emergency care include sponsoring nurses to pursue emergency nursing care as a specialty and provision of overtime allowances or lunch to nurses in EDs. Currently, there is only one university in Ghana offering emergency care nursing [33]. Emergency nursing should be introduced in other universities in Ghana in order to increase the number of emergency nurses in Ghana. A similar study could be extended to other regions of Ghana in order to compare results.
